# The mycorrhizal pathway of zinc uptake contributes to zinc accumulation in barley and wheat grain

**DOI:** 10.1186/s12870-019-1741-y

**Published:** 2019-04-10

**Authors:** Antonio Coccina, Timothy R. Cavagnaro, Elisa Pellegrino, Laura Ercoli, Michael J. McLaughlin, Stephanie J. Watts-Williams

**Affiliations:** 1 Institute of Life Sciences, Scuola Superiore Sant’Anna, Piazza Martiri della Libertà 33, 56127 Pisa, Italy; 2 The School of Agriculture, Food and Wine, and the Waite Research Institute, The University of Adelaide, PMB 1, Glen Osmond, South Australia 5064 Australia; 3 Australian Research Council Centre of Excellence in Plant Energy Biology, The University of Adelaide, Glen Osmond, South Australia Australia

**Keywords:** Arbuscular mycorrhizal fungi, Barley (*Hordeum vulgare*), Radioisotope tracing, Wheat (*Triticum aestivum*), Yield, Zinc nutrition

## Abstract

**Background:**

Increasing zinc (Zn) concentrations in crops is important for alleviation of human Zn deficiency. Arbuscular mycorrhizal fungi (AMF) contribute to plant Zn uptake, but their contribution to Zn in the edible portion of crops has not yet been investigated. This study aimed to quantify the mycorrhizal pathway of Zn uptake into grain of wheat and barley under varying soil Zn availabilities. Bread wheat (*Triticum aestivum*) and barley (*Hordeum vulgare*) were grown in pots with a hyphal compartment containing ^65^Zn. Plants were inoculated with *Rhizophagus irregularis* and grown at three soil Zn concentrations. Radioactive Zn in grain and straw was measured and the contribution of AMF to Zn uptake was calculated.

**Results:**

The mycorrhizal pathway of Zn uptake contributed up to 24.3% of total above-ground Zn in wheat, and up to 12.7% of that Zn in barley. The greatest contribution by the mycorrhizal pathway was observed in barley at the lowest Zn addition, and in wheat at the highest one. In addition, grain yield of bread wheat was increased by AMF.

**Conclusions:**

These results suggest that AMF have a substantial role in uptake of Zn into cereals, and the proportional contribution by the MPU is dependent on plant species, as well as available soil Zn.

**Electronic supplementary material:**

The online version of this article (10.1186/s12870-019-1741-y) contains supplementary material, which is available to authorized users.

## Background

Zinc (Zn) malnutrition is a major global health problem for people relying on cereal-based foods as their major source of energy and minerals [4]. It was estimated that 17.3% of the world’s population is at risk of inadequate dietary Zn intake [51]. This problem is especially pertinent in the regions where the plant-available (rather than total) Zn in soils is low [6]. Consequently, finding solutions for increasing Zn concentrations in the edible portions of crop plants is an important hurdle to improve food quantity and quality, especially in the context of an increasing global human population and a changing climate [31].

The lack of sufficient Zn in plants can affect the synthesis and function of a wide range of macromolecules, and decrease the yield and quality of crops as a consequence [2, 6, 9, 49]. Furthermore, foods produced from Zn-deficient crops (considered to be < 15 and < 20 mg Zn kg^− 1^ dry mass, in grains and shoots, respectively) may result in human Zn deficiency, which can in turn have an impact on human wellbeing by reducing the body immune functions, and increasing the risk of growth stunting in children or the risk of adverse pregnancy outcomes in women [2, 19].

Plants mainly acquire Zn from the soil in the form of free ions (Zn^2+^ and ZnOH^+^). Numerous soil edaphic factors limit Zn phytoavailability, including: low total Zn concentration, high CaCO_3_, high organic matter contents (> 3%), neutral or alkaline pH, low redox conditions, high concentration of ligands capable of forming organo-Zn complexes, and high micronutrient or macronutrient (especially P) concentrations [2, 24, 26]. However, Zn can also be toxic for the plants when present in excess in soil [32]. Increasing plant acquisition of Zn in Zn-deficient soils has been studied previously [6, 7, 42, 43, 50]. For example, in Southeast Asia, the interdisciplinary program HarvestPlus continues to develop and release new wheat and other crop varieties that have been bred to accumulate higher grain Zn concentration [37].

Arbuscular mycorrhizal fungi (AMF) can form associations with the roots of about 80% of terrestrial plant species, and exchange soil-derived nutrients for plant-derived photosynthates and lipids [22, 39]. Since AMF have the ability to improve the nutrition of the host plant through increased uptake of soil mineral nutrients, their potential as natural fertilizers (biofertilizers) is increasingly recognised; this is especially relevant for the uptake of relatively immobile nutrients (e.g. P, Zn, Fe, Cu, K) from the nutrient depletion zones that can form around roots [33, 34, 46]. Moreover, AMF can alleviate heavy metal toxicity in the host plants and help to tolerate to high metal concentrations in the soil [18, 27, 30, 41].

Plants colonized by AMF have two soil nutrient uptake pathways: (1) directly via the root epidermis (direct pathway of uptake; DPU), and (2) via fungal structures that form the mycorrhizal pathway of uptake (MPU) [40]. Using ^65^Zn, Jansa et al. [20] quantified the MPU contribution to plant Zn by the proportion of the labelled nutrient added and transported into the shoots of maize plants inoculated with the AMF *Glomus intraradices* (renamed to: *Rhizophagus irregularis*). More recently, Watts-Williams et al. [48] used ^65^Zn to quantify the total amount of Zn and the relative contribution (%) of Zn delivered via the MPU and DPU, respectively, in shoots of tomato plants inoculated with *R. irregularis*. In that study, the greatest amount of Zn delivered via the MPU to the shoots was 21.7 μg at the medium Zn concentration treatment (DTPA-extractable Zn 9.0 μg g^− 1^), and the highest relative contribution of the MPU was up to 24.2% at the low Zn concentration treatment (soil DTPA-extractable Zn 1.0 μg g^− 1^).

It is likely that values of contribution by AMF to plant Zn uptake are highly dependent on the host plant species, as is the case for P uptake [40]. Therefore, it is important to quantify the MPU for Zn in important crop species. Cereal crops represent a major source of minerals and protein in the developing world, but around the 50% of soils where cereals are cultivated are considered Zn-deficient [2, 6]. To our knowledge, there are no studies that have directly measured the contribution of the Zn MPU to the edible portion of any cereal species, thus we designed this study to test the following specific aims:

1. To quantify the contribution of the MPU to Zn uptake in bread wheat and barley, and in particular, to the grains;
2. To investigate whether contribution to plant Zn via the MPU is modified under a range of soil Zn concentrations;
3. To study the effects of AMF inoculation on plant yield and Zn concentration in bread wheat and barley.

To test these aims, we undertook a glasshouse study in which radioactively labelled Zn was used to quantify the MPU for two cereal crops (bread wheat and barley) at three different soil Zn availabilities.

## Results

### Arbuscular mycorrhizal fungal colonization

**Fig. 1:**
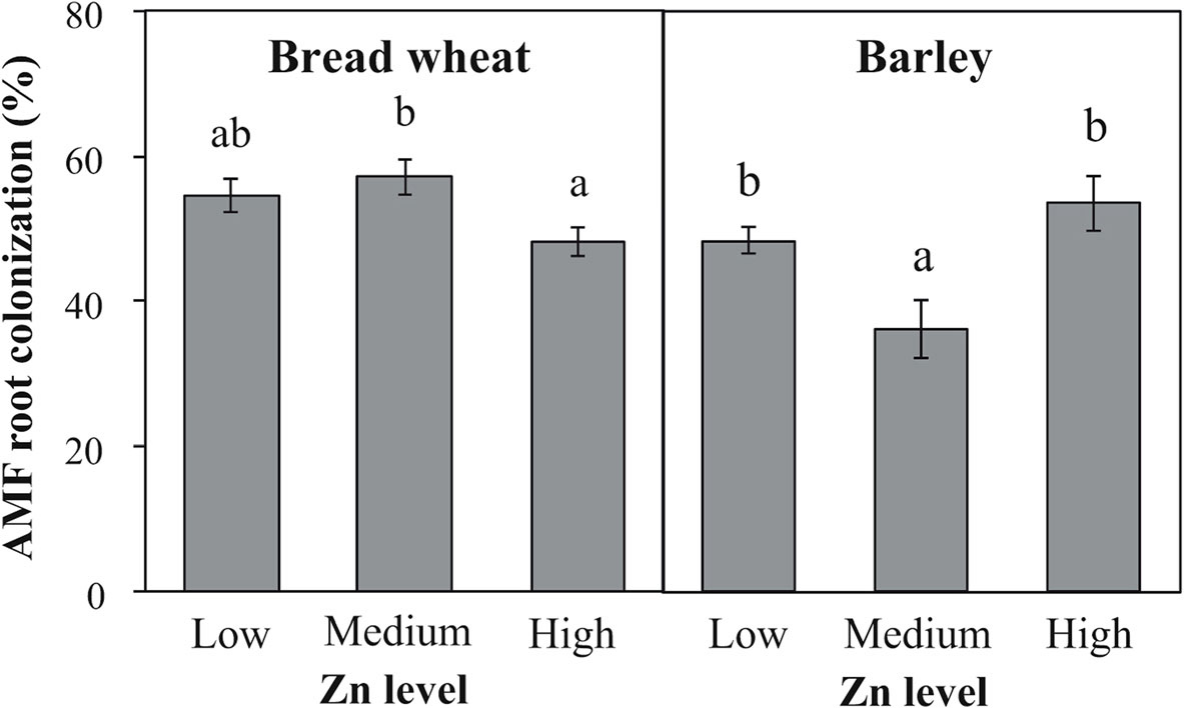
Effect of Zn application on AMF root colonization of bread wheat and barley sampled at physiological maturity (Zadoks growth stage 90). Values are mean ± SEM, *n* = 5. Means followed by the same letter are not significantly different (*P* > 0.05, see text for further details)

At physiological maturity (GS90), roots of bread wheat and barley inoculated with *Rhizophagus irregularis* were well colonized (mean values of 53 and 46%, for bread wheat and barley, respectively) and the percentage of colonization was significantly affected by Zn application (Additional file 1: Table S1; Fig. 1). In bread wheat, mycorrhizal colonization decreased by 16% with increasing soil Zn concentration from Low/Medium Zn to High Zn. By contrast, in barley AMF root colonization was higher at Low Zn and High Zn (mean value: 51%) than at Medium Zn (36%).

### Plant growth, yield and yield components

For bread wheat and barley, the plant biomass, yield, and yield components were differently affected by AMF inoculation and Zn application (Table 1). In bread wheat, above-ground biomass (grain + straw + chaff) was greater at Medium Zn than at Low Zn or High Zn, and did not vary with AMF inoculation (Additional file 1: Table S2).

**Table 1:** *P*- values of two way ANOVA evaluating the effect of AMF inoculation with *Rhizophagus irregularis* (AMF Inoc) and three soil Zn levels (Zn application) on above ground plant parts, grain yield and yield components, Spike Fertility Index, and P content and Zn concentrations and content in grain and straw of bread wheat and barley sampled at physiological maturity (Zadoks growth stage 90)

Factors^a^	Above ground biomass	Grain biomass	Straw biomass	Chaff biomass	No. of kernels spike^−1^	Mean kernel weight	Spike Fertility Index	Grain P content	Straw P content	Grain Zn concentration	Grain Zn content	Straw Zn concentration	Straw Zn content
**Bread wheat**													
AMF Inoc^b^	0.489^d^	**< 0.001**	0.741	**< 0.001**	**< 0.001**	0.787	**< 0.001**	**0.001**	**< 0.001**	**< 0.001**	**0.006**	**0.001**	**< 0.001**
Zn application^c^	**0.018**	0.475	**0.003**	0.815	0.365	**0.047**	0.548	0.317	**< 0.001**	**< 0.001**	**< 0.001**	**< 0.001**	**< 0.001**
AMF Inoc x Zn application	0.246	0.124	0.444	0.263	0.143	0.079	0.132	**0.021**	**0.001**	**< 0.001**	**0.004**	**0.008**	0.190
**Barley**													
AMF Inoc	0.453	0.109	0.085	0.077	0.525	**0.025**	0.071	0.193	**0.007**	0.2302	0.067	0.2461	0.416
Zn application	0.341	0.840	0.217	0.576	0.825	0.718	0.470	0.717	0.625	**< 0.001**	**< 0.001**	**< 0.001**	**< 0.001**
AMF Inoc x Zn application	0.637	0.291	0.389	0.695	**0.004**	0.659	0.757	0.333	0.493	0.2964	0.100	0.0893	0.295

^a^AMF Inoc and Zn application are used as fixed factors

^b^AMF inoculation levels: mock inoculated and inoculated with *Rhizophagus irregularis*

^c^Soil zinc levels: 0, 20 and 75 mg Zn kg^− 1^ soil in the form of ZnSO_4_

^d^In bold statistically significant values (*P* ≤ 0.05) (No of replicates = 5)

**Fig. 2:**
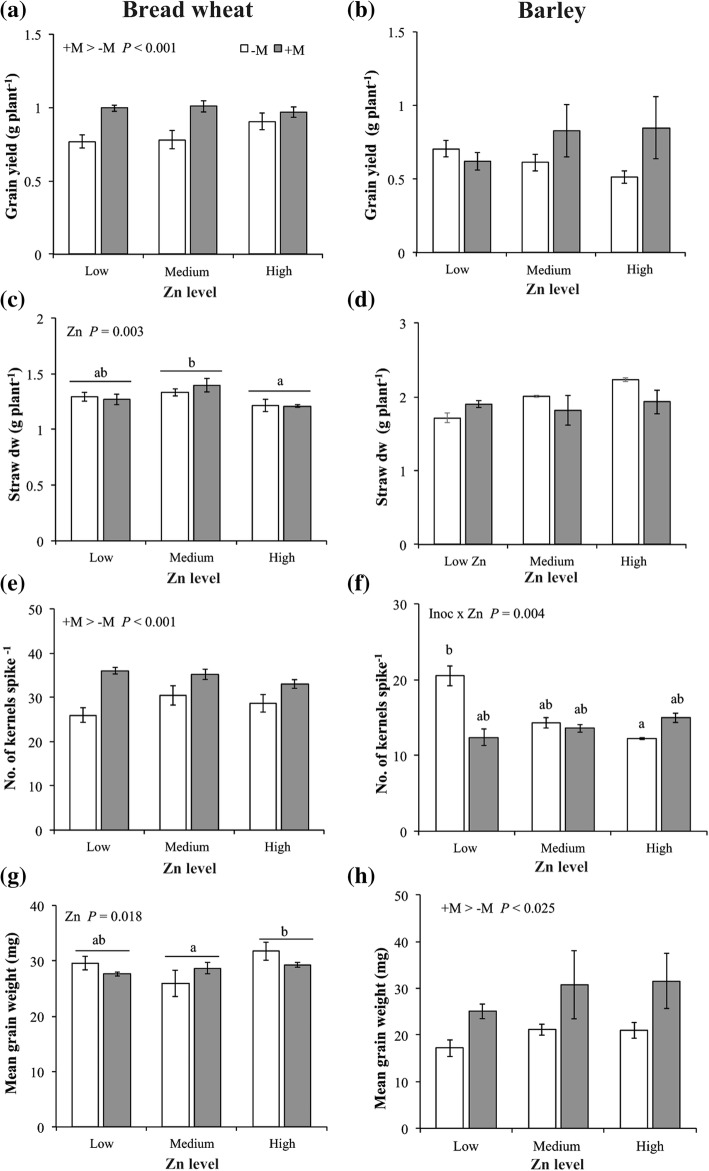
Effect of Zn application and AMF inoculation with *Rhizophagus irregularis* (grey bars) or mock-inoculation (white bars) on grain yield, straw dry weight (dw), number of kernels per spike, and mean kernel weight of bread wheat (a, c, e, g, respectively) and barley (b, d, f, h, respectively). Plants were sampled at physiological maturity (Zadoks growth stage 90). Values are mean ± SEM, *n* = 5. Means followed by the same letter are not significantly different (*P* > 0.05, see text for further details)

In bread wheat, the grain yield, chaff, number of kernels per spike, and Spike Fertility Index (SFI) were each significantly affected by AMF inoculation. Specifically, grain yield was 21% greater in the inoculated plants (+M) than in the control plants (−M) (0.99 g and 0.82 g plant^− 1^, respectively; Fig. 2a), and number of kernels per spike, and SFI were 23 and 73% higher in the +M plants, respectively (Fig. 2e). By contrast, chaff was 28% lower in +M than in –M plants (Additional file 1: Table S2). Straw biomass (Fig. 2c) and mean kernel weight (MKW; Fig. 2g) were not affected by AMF inoculation, but were modified by Zn application, with values decreasing by 8 and 3%, respectively, from Low/Medium Zn to High Zn.

In barley, neither AMF inoculation nor Zn application affected above ground biomass, grain yield, straw, chaff or SFI (Table 1; Fig. 2b,d; Additional file 1: Table S3). Among yield components, number of kernels per spike was affected by the interaction between AMF inoculation and Zn application, and MKW by AMF inoculation only. The number of kernels per spike was 52% higher at Low Zn in plants of barley not inoculated compared to all other treatments (Fig. 2f). Moreover, the MKW was 37% higher in +M than in –M barley plants (Fig. 2h).

### Plant zinc and phosphorus nutrition

**Fig. 3:**
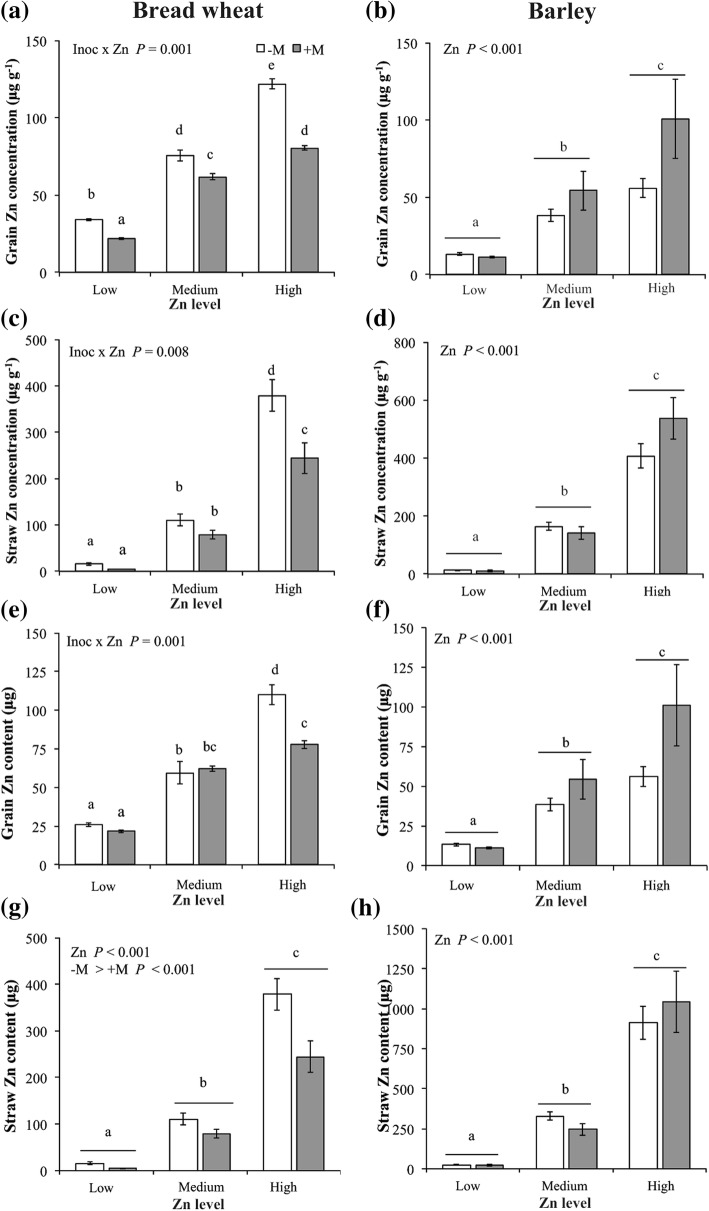
Effect of Zn application and AMF inoculation with *Rhizophagus irregularis* (grey bars) or mock-inoculation (white bars) on Zn concentration in grain and straw (**a**, **c**) and on Zn content in grain and straw (**e**, **g**) in bread wheat, and Zn concentration in grain and straw (**b**, **d**) and on Zn content in grain and straw (f, h) in barley. Plants were sampled at physiological maturity (Zadoks growth stage 90). Values are mean ± SEM, *n* = 5. Means followed by the same letter are not significantly different (*P* > 0.05, see text for further details)

In bread wheat, both grain and straw Zn concentrations were affected by the interaction between AMF inoculation and Zn application (Table 1); in the grain, the –M plants had higher Zn concentrations than the +M at each Zn level, respectively (Fig. 3a), and grain Zn concentration ranged from 28.0 μg Zn g^− 1^ (mean Low Zn) to 101.18 μg Zn g^− 1^ (mean High Zn). In the straw component, there was a sharp increase in Zn concentration from Low Zn (mean 7.8 μg Zn g^− 1^) to High Zn (mean of 256.3 μg Zn g^− 1^), and at High Zn, concentrations were higher in the –M than the +M plants (Fig. 3c).

The Zn concentration of barley grain and straw were each affected by the main effect of Zn application, whereby concentrations increased from Low Zn to High Zn (Table 1). For the straw component, the increase in mean Zn concentration from Low Zn to High Zn was marked: from 11.73 μg Zn g^− 1^ up to 472.6 μg Zn g^− 1^ (Fig. 3d). The grain Zn concentrations were kept within a much smaller range, from 19.14 μg Zn g^− 1^ up to 114.16 μg Zn g^− 1^ (Fig. 3b).

The Zn content of bread wheat grain was affected by the interaction between AMF inoculation and Zn application, while Zn content in straw was affected by the main effects of AMF inoculation and Zn application (Table 1). Grain Zn content increased 1.5 fold from Low Zn to Medium Zn irrespective of AMF inoculation (on average 24.0 versus 60.8 μg plant^− 1^), whereas it increased by 85% from Medium Zn to High Zn only in the -M plants (59.5 versus 110 μg plant^− 1^) (Fig. 3e). The accumulation of Zn in the at High Zn was lower in the +M plants than in the –M plants (77.7 versus 110.0 μg plant^− 1^). A similar trend was observed in the straw Zn content where the +M plants were lower than that of the –M plants (mean 109.2 versus 168.3 μg plant^− 1^, pooling Zn treatments; Fig. 3g).

As for concentrations, the Zn content in barley grain and straw was increased by Zn application (Fig. 3f). Zinc content in grain increased by 280 and 69% following the increase of Zn application from Low Zn to Medium Zn, and again from Medium Zn to High Zn, respectively (12.2, 46.4 and 78.4 μg plant^− 1^ in Low, Medium and High Zn, respectively). A similar trend was found for straw Zn content (22.5, 287.9 and 977.8 μg plant^− 1^ in Low, Medium and High Zn, respectively; Fig. 3h).

In bread wheat, P content in grain and straw were affected by the interaction between AMF inoculation and Zn application (Table 1). AMF inoculation lead to a significant increase of grain P content at Low Zn and Medium Zn (+ 28% and + 25%, respectively), while at High Zn level AMF inoculation did not modify P content in grain (Additional file 1: Table S2). At High Zn, the P content in grain of both +M and -M plants (3.5 mg plant^− 1^) was similar to the values detected in +M plants grown at lower Zn availabilities (3.7 mg plant^− 1^). In the -M plants, the P content in straw decreased from 2.9 mg per plant at Low Zn and Medium Zn level to 2.1 mg per plant at High Zn (rate of decrease: 26%). Inoculation by AMF decreased P content in straw at all Zn applications, but the rate of decrease was different according to Zn application, ranging from 44% at Low Zn and Medium Zn to 29% High Zn.

In barley, the P content in grain did not vary according to AMF inoculation or Zn application, whereas P content in straw was affected by AMF inoculation (Table 1). In detail, P content in straw was 26% higher in the –M than the +M plants (Additional file 1: Table S3).

### Mycorrhizal pathway contribution to zinc uptake in grain and straw

The activity of ^65^Zn in the -M plants was minimal and did not differ from background activity (*P* > 0.05; data not shown), confirming that there was no loss of ^65^Zn out of the HCs. By contrast, the activity in the +M plants was from seven to nine orders of magnitude higher than background activity (*P* < 0.05; data not shown). For this reason, and for the absence of mycorrhizal colonisation in the -M plants, we excluded the data from mock-inoculated plants in the following calculations and analyses.

**Fig. 4:**
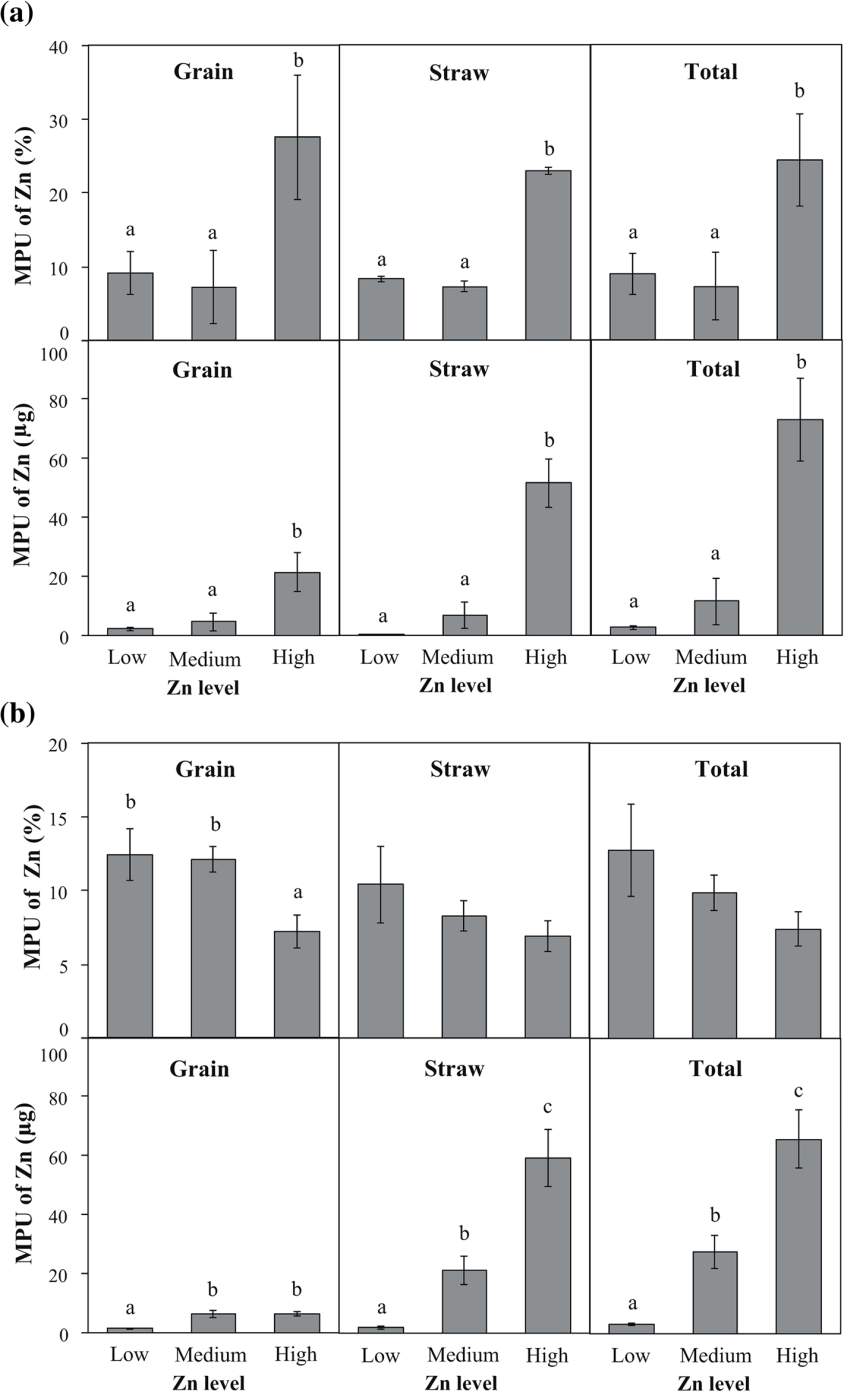
Effect of Zn application on mycorrhizal pathway of Zn uptake (MPU of Zn) in grain, straw and total plant (grain + straw) (% and μg) of bread wheat (a) and barley (b). Values are mean ± SEM, n = 5. Means followed by the same letter are not significantly different (*P* > 0.05, see text for further details)

In bread wheat, the mycorrhizal pathway of uptake (MPU) of Zn in grain, straw and total plant (grain + straw) expressed both as % Zn, and as μg Zn, was significantly affected by Zn application (Table 2). The percentage of Zn delivered by MPU in grain did not change between Low Zn and Medium Zn (mean value: 8.2%), whereas it was significantly higher at High Zn (27.5%) (Fig. 4a). Similarly, the percentage of Zn delivered by MPU to the straw and total plant did not change between Low Zn and Medium Zn (7.8% and 8.1%, respectively), whereas it was significantly higher at High Zn (22.9% and 24.3%, respectively). Regarding Zn contents of the grain, straw and total plant, the amount of Zn delivered by MPU was similar between Low Zn and Medium Zn, with mean values of 3.4, 3.6 and 7.0 μg Zn μg Zn plant^− 1^, respectively. At High Zn, the amount of Zn delivered by MPU to the grain, straw and total plant (21.4, 51.6 and 73.0 μg Zn plant^− 1^, respectively) was significantly higher than the amounts delivered at Low and Medium Zn.

**Table 2:** *P*-values of one-way ANOVA evaluating the effect of three soil Zn levels on mycorrhizal pathway of Zn uptake (MPU of Zn) in grain, straw and total plant (grain+straw) (%; μg) and on direct pathway of uptake (DPU of Zn) in grain, straw and total plant (grain+straw) (μg) of bread wheat and barley inoculated with *Rhizophagus irregularis*. Plants were sampled at physiological maturity (Zadoks growth stage 90)

Parameters^a^	Wheat	Barley
MPU of Zn in grain (%)	**0.046** ^b^	**0.016**
MPU of Zn in straw (%)	**0.024**	0.469
MPU of Zn in total plant (%)	**0.033**	0.231
MPU of Zn in grain (μg)	**< 0.001**	**0.002**
MPU of Zn in straw (μg)	**< 0.001**	**< 0.001**
MPU of Zn in total plant (μg)	**< 0.001**	**< 0.001**
DPU of Zn in grain (μg)	**< 0.001**	**0.010**
DPU of Zn in straw (μg)	**< 0.001**	**< 0.001**
DPU of Zn in total plant (μg)	**< 0.001**	**< 0.001**

^a^Soil zinc levels: 0, 20 and 75 mg kg^− 1^ soil in the form of ZnSO_4_.7H_2_O

^b^In bold statistically significant values (*P* ≤ 0.05). (No of replicates = 5)

**Fig. 5:**
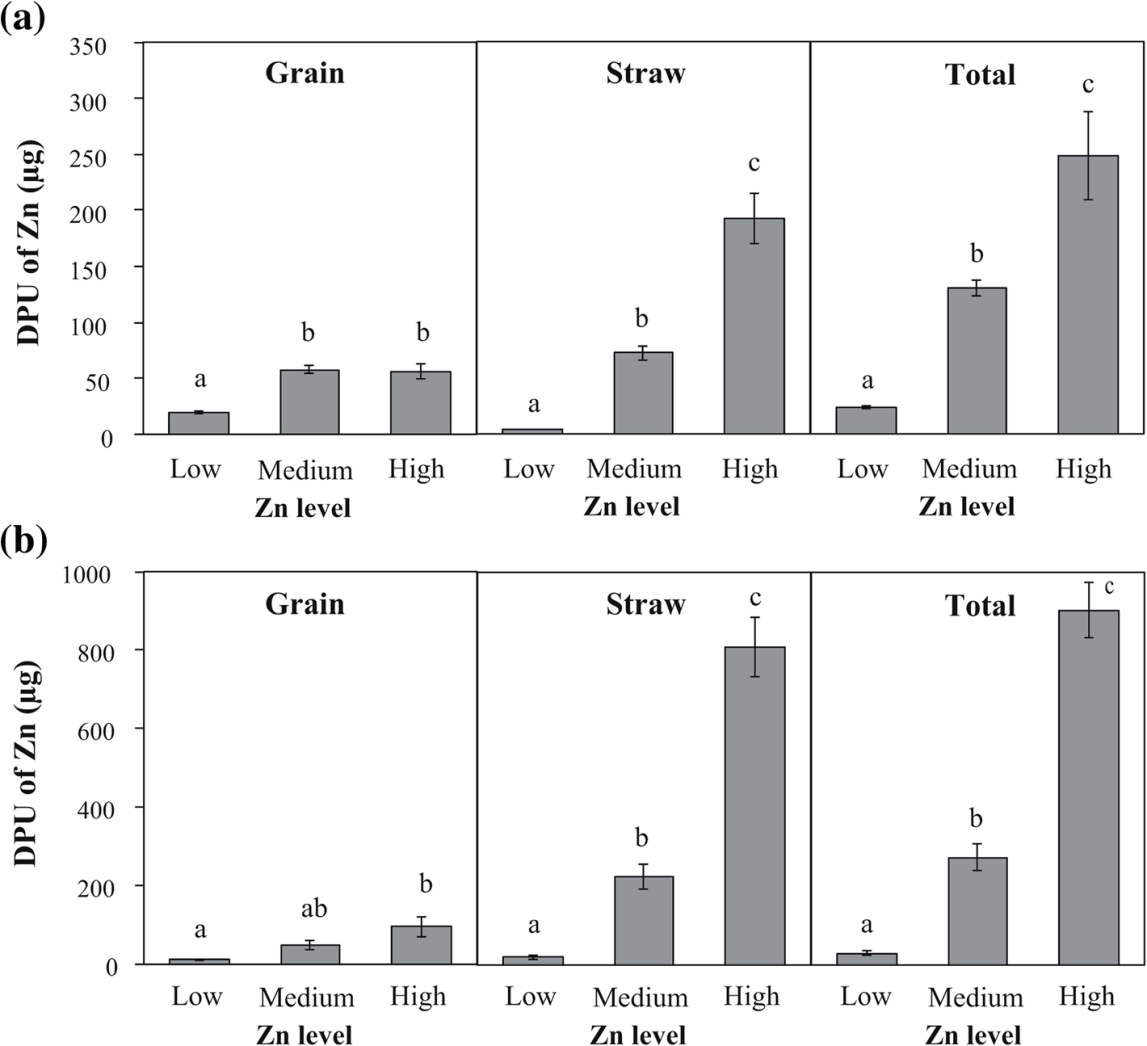
Effect of Zn application on direct pathway of uptake (DPU of Zn) in grain, straw and total plant (grain + straw) (μg) of bread wheat (**a**) and barley (**b**). Values are mean ± SEM, n = 5. Means followed by the same letter are not significantly different (*P* > 0.05, see text for further details)

The direct pathway of Zn uptake (DPU) in bread wheat was significantly affected by Zn level (Table 2). The amount of Zn delivered by DPU in grain did not vary between Medium Zn and High Zn (mean 57.1 μg Zn plant^− 1^), but was significantly lower at Low Zn (19.8 μg Zn plant^− 1^) (Fig. 5a). The amount of Zn delivered by the DPU to the straw and total plant progressively increased with the increase of Zn application. At High Zn, the amount of Zn delivered by DPU to the straw and total plant was 46 and 9 fold higher than at Low Zn, respectively (straw: 4.1 versus 192.2 μg Zn plant^− 1^; total plant: 23.9 versus 248.6 μg Zn plant^− 1^).

In barley, the MPU of Zn into the grain, expressed as percentage of total Zn content, was significantly affected by Zn application (Table 2); at Low and Medium Zn, the MPU was similar with mean value of 12.3%, and decreased to 7.2% at High Zn (Fig. 4b). When the MPU was expressed in μg Zn plant^− 1^, a different pattern of response to Zn application was observed. The MPU for Zn content in grain, straw and total plant were significantly affected by Zn application (Table 2). In grain, MPU increased by 360% from Low Zn to Medium/High Zn (1.4 versus 6.5 μg Zn plant^− 1^). The MPU to straw and total plant progressively increased from Low through to High Zn. At the highest Zn level, the amount of Zn delivered by the DPU in straw and the total plant was 33- and 20-fold higher than at Low Zn, respectively (straw: 1.8 versus 59.2 μg Zn plant^− 1^; total plant: 3.2 versus 65.8 μg Zn plant^− 1^).

The DPU of Zn to the grain, straw and total plant in barley was significantly affected by Zn application (Table 2). The amount of Zn delivered by DPU to the grain significantly increased between Low Zn and High Zn (9.8 versus 94.3 μg Zn plant^− 1^); the DPU to straw and the total plant also progressively increased with increasing Zn application (Fig. 5b). At High Zn, the amount of Zn delivered by DPU to the straw and total plant was 46- and 9-fold higher than at Low Zn, respectively (straw: 19.3 versus 809.3 μg Zn plant^− 1^; total plant: 29.0 versus 903.6 μg Zn plant^− 1^).

## Discussion

Here we aimed to fill an important knowledge gap: quantification of the contribution by the mycorrhizal pathway of uptake to Zn in the grain of bread wheat and barley, under a range of soil Zn concentrations. We also aimed to study the effects of AMF inoculation on grain yield and nutrition of bread wheat and barley. We discovered that the mycorrhizal pathway of Zn uptake contributed up to 24.3% of the Zn in bread wheat, and up to 12.7% of the Zn in barley; the contribution to Zn via the mycorrhizal pathway was highly dependent on the soil Zn concentration. Results are now discussed in the context of the role of mycorrhizal impacts on yield and nutritional quality of two of the world’s major cereal crops.

### Arbuscular mycorrhizal fungal colonization

Both bread wheat and barley plants were well colonized by *R. irregularis,* which is in line with previous work of Al-Karaki and Al-Omoush [1] in wheat, and of Watts-Williams and Cavagnaro [46] using the same barley cultivar. The effects of increasing soil Zn concentration on mycorrhizal colonization in bread wheat were negative, and variable in barley. Variable impacts of soil Zn supply for mycorrhizal colonisation have been reported in the literature. For example, the colonisation of wild tobacco roots by AMF increased with increasing Zn supply in one study [3] whereas in the same soil as the present study, Cavagnaro et al. [7] and Watts-Williams et al. [48] in tomato, observed no impact of soil Zn concentration on percent colonisation of roots due to Zn addition in soil, and using the same barley cultivar, Watts-Williams and Cavagnaro [46] found decreased mycorrhizal colonisation with increasing soil Zn.

### Cereal growth, yield, and yield components

Inoculation with *R. irregularis* led to increased grain yield in bread wheat but not in barley, in this study. This is consistent with a recent meta-analysis that reported a positive effect of AMF on wheat yield (17% increase), but only a neutral effect on barley yield across many field- and glasshouse-based trials [54]. Pellegrino et al. [35] present another meta-analysis showing wheat yield increased due to AMF field inoculation by 20%, from field trials across the world. Consistently, similar yield increases (18%) were also observed in several bread wheat genotypes field-inoculated with *R. irregularis* [36]. Mycorrhizal plants in the present study also had increased number of grains per spike and decreased chaff, which are desirable traits for improving yield [15, 16]. The increased grain yield and yield components associated with the formation of AMF in bread wheat may be due to changes in allocation of P in colonized plants, since the content of P in grains was greater in inoculated plants compared to the mock-inoculated ones. It must be noted however, that the sandy substrate, small pot size, and exclusive inoculation with *R. irregularis* that were used in this study, are not representative of a field situation, and thus the opportunity to extrapolate the results to a field situation are limited.

### Cereal zinc nutrition

One of the benefits of forming AMF for plants is improved Zn nutrition [9]. Numerous studies, including a meta-analysis, have highlighted that inoculation with AMF can lead to increased Zn concentration in the edible portion of crops including the grain [17, 24, 46]. In the present study, however, grain Zn concentration was actually reduced in the mycorrhizal bread wheat plants, and unaffected by inoculation in the barley plants. This reduction in Zn concentration in bread wheat was compensated by increased grain yield in the mycorrhizal plants, leading to similar total Zn uptake regardless of mycorrhizal inoculation. However, the concentration of grain Zn is the important factor for improved biofortification outcomes [5] and the consumed products of the grain [13]. Thus, in the present study, AMF inoculation did not present potential for biofortification of the bread wheat or barley.

Although the soil Zn concentration reached very high levels in the High Zn treatment (DTPA-Zn of 41.4 mg Zn kg^− 1^), we did not observe a positive biomass response to mycorrhizal inoculation in this treatment to suggest that the classic “protective effect” of mycorrhizas at toxic Zn levels was active, as has been shown in other plant species [10, 11, 23, 47]. In other studies, the protective effect of AMF was observed in *M. truncatula* at 17 mg Zn kg^− 1^ soil (DTPA-extractable Zn) [49], tomato at 25 mg Zn kg^− 1^ soil [7], and in red clover after the addition of 50 mg Zn kg^− 1^ soil [10]. However, in the present study at the High Zn level, there appeared to be a protective effect of AMF in terms of relative Zn allocation to the straw or grain, rather than improvement in biomass accumulation. Importantly, the critical value for the protective effect to engage will be dependent upon soil cation exchange capacity, which affects the bioavailable Zn that is ‘active’ for the expression of soil toxicity [45].

### The mycorrhizal pathway of zinc uptake

Our results demonstrated that a substantial proportion of total plant Zn can be obtained via the mycorrhizal pathway of uptake in cereals. We discovered that the mycorrhizal pathway of Zn uptake (MPU) contributed up to a quarter of the Zn in bread wheat, and up to an eighth of the Zn in barley. Our finding that the mycorrhizal pathway of Zn uptake is important to plant Zn nutrition in these cereal crops is in agreement with earlier work on a range of plant species.

Our research demonstrated in bread wheat and barley that AMF contribution to plant Zn increased with increased available soil Zn in terms of absolute contribution (μg Zn). This is in contrast to data from tracing the MPU of Zn in tomato, where absolute Zn contributions remained constant across three different soil Zn concentrations [48]. In the present study, it appears that AMF are unable to regulate the amount of Zn entering the plant via the MPU, even when Zn is in excess and potentially detrimental to the plant. This may at least partially explain why we do not see a protective effect of AMF (i.e., increased biomass) in the barley and wheat plants. Although there were differences in proportional contribution via the MPU between wheat and barley at high Zn concentrations, it is worth noting that the contribution in μg via the mycorrhizal pathway was almost identical between the two species (70 and 66 μg Zn in bread wheat and barley, respectively). Given that the same AMF species contributed the same amount of Zn to two different plant species, it remains to be seen whether different species of AMF confer different contributions via the MPU. Such studies would inform on whether the colonising AMF species have more of an influence on MPU activity than the species of host plant.

The influence of different species of AMF on enhancing Zn plant nutrition of wheat was observed by Daei et al. [14]. Barley plants inoculated with *Glomus contrictus* or *Glomus fasciculatus* showed an increased Zn uptake, whereas plants inoculated with *Glomus margarita* did not differ from control plants in Zn uptake [21]. Moreover, the response of the combination of plant species/genotype and AMF species/isolate would be modulated by other soil properties such as pH, N availability, exchangeable calcium and base saturation, and climatic conditions [12, 17, 29]. Thus it is likely that the greater AMF-mediated Zn uptake in bread wheat compared to barley depends on the higher compatibility and efficiency of the wheat variety with the isolate of *R. irregularis* used.

In bread wheat and barley, the partitioning of the AMF-mediated Zn uptake within plant tissues changed with Zn availability in soil. At the lowest Zn level, 86% and 44% of MPU Zn was allocated to grain in bread wheat and barley, respectively. This proportion decreased to 29% and 10% at the highest Zn level, in bread wheat and barley, respectively. It is possible that this is a result of plants buffering the grain from accumulating high amounts of Zn under toxic soil Zn conditions, so as to ‘protect’ the viability of the seed, as was observed in the same barley cultivar previously [46].

## Conclusions

Here we have quantified for the first time, the mycorrhizal pathway of Zn uptake to the edible portion of two important cereal crop species. Mycorrhizal fungi can contribute substantially to the Zn nutrition of cereal crops, but that the contribution cannot be generalised between bread wheat and barley. Furthermore, the contribution by the mycorrhizal pathway was highly dependent on soil Zn availability, and the relative allocation of Zn between grain and straw acted independently of mycorrhizal inoculation. The greatest contribution by the mycorrhizal pathway was observed at the lowest Zn addition in barley, and in contrast, the highest Zn addition in bread wheat. Additionally, the increase of grain yield in mycorrhizal bread wheat by 21.2% highlights the potential benefits of mycorrhizal fungi for yield, but the Zn concentration of bread wheat grain was reduced by mycorrhizal inoculation. These results suggest that the role played by AMF on Zn uptake depends on the functional compatibility between AMF isolate and inoculated cereal species. However, future studies using different cultivars of barley and varieties of bread wheat will provide more information on this hypothesis.

## Methods

### Preparation of hyphal compartments, soils, and plants

A compartmented pot design was used to quantify the contribution of AMF to Zn uptake in bread wheat and barley, following Watts-Williams et al. [48] (Additional file 1: Figure S1). The hyphal compartments (HCs) were small plastic vials packed with 40 g soil that had been mixed thoroughly with 428 ± 12 kBq of ^65^Zn in the form of ^65^ZnCl_2_ (Perkin-Elmer, U.S.A.), and then a 10 g layer of unlabelled soil. The HCs were capped with a 25 μm nylon mesh which allowed the penetration of the AMF hyphae but not of the plant roots. The HC lids were then tightly sealed with electrical tape to prevent the penetration of plant roots. A HC was placed in each pot in the same position, with the nylon mesh side facing towards the center of the pot. In addition, plant-free pots (*n* = 9) containing one HC each were prepared as controls to measure soil Zn concentration and ^65^Zn activity at three different Zn concentration treatments (Low, Medium, and High Zn, replicated three times). Plastic 1 L pots were filled with 1.4 kg of a 9:1 (*w/w*) sand/soil mixture containing 140 g of *R. irregularis* WFVAM10 inoculum, or of mock inoculum performing as a non-mycorrhizal control. The *R. irregularis* WFVAM10 is synonymous with DAOM 181602, an earlier voucher number for DAOM 197198, formerly named *Glomus intraradices*, originally from subcultured from an axenic culture on transformed roots obtained from Professor J. A. Fortin, University of Montreal, Canada. The *R. irregularis* inoculum was added as a mix of dry soil, fungal spores and external hyphae, and root fragments of Marigold (*Tagetes patula*) pot cultures produced on-site. The control, a mock inoculum, was a mixture of dry soil and root fragments of Marigold pots that had not been inoculated with AMF; the inoculum was grown on the same sand/soil substrate that makes up the other 90% of the pot, so the proportional volume was the same as for mass (one tenth, or 100 mL in the 1 L plastic pot). The soil was collected from the Mallala region of South Australia [48], and the chemical characteristics of the soil were as follows: pH_1:5 (water)_ 7.1; plant available (Olsen) P 16 mg kg^− 1^, and DTPA- extractable Zn 0.65 mg kg^− 1^ [25]. The soil was firstly sieved to < 2 mm and the sand/soil mixture was sterilised by autoclaving (121 °C for 25 min, twice). The sand/soil mixture, which is referred to as ‘soil’ hereafter, was amended with 20 or 75 mg Zn kg^− 1^ soil, in the form of ZnSO_4_·7H_2_O solution. These treatments are henceforth referred to as Medium Zn and High Zn treatments, respectively. After the application of Zn to soil, the plant-available (DTPA-extractable) Zn [25] concentrations were 12.9 ± 1.2 mg Zn and 41.4 ± 2.7 mg Zn kg^− 1^ soil, for the Medium Zn and High Zn treatments, respectively. In addition, the Low Zn treatment had no Zn application, and its DTPA-extractable Zn concentration was 0.19 ± 0.07 mg Zn kg^− 1^ soil. All pots were fertilized with 25 mg of anhydrous CaHPO_4_ kg^− 1^ soil in order to stimulate plant growth in the otherwise low nutrient soil, without inhibiting AMF colonization.

Seeds of bread wheat (*Triticum aestivum* L.) cv. Axe (obtained from Australian Grain Technologies), and two-rowed barley (*Hordeum vulgare* L.) cv. Compass (originally obtained from the Barley Breeding Program, University of Adelaide), were surface-sterilised by immersion in 10% sodium hypochlorite solution for 10 min, and rinsed three times with deionized (DI) water. Seeds were pre-germinated on moist filter paper in a Petri dish for 2 days at 25 °C in the dark, and two pre-germinated seeds were planted into each pot. After one week the seedlings were thinned to one per pot. Plants were grown in a controlled environment glasshouse at the University of Adelaide’s Waite campus, during the months May–July 2017. Over this period, glasshouse mean maximum temperature was 21.2 ± 0.14 °C, and mean minimum temperature was 8.2 ± 0.30 °C. All pots were watered twice per week with reverse osmosis (RO) water, and from week two were fertilised once per week with a 1/10 strength modified Long Ashton solution (omitting P and Zn) [8].

The plants were additionally fertilized with a total of 50 mg of nitrogen (N) per pot as NH_4_NO_3_ over the course of the experiment: 25 mg N was applied at pseudo-stem stage (Zadoks growth stage (GS) 30), and 25 mg N at flag leaf sheath opening stage (GS47) [52]. The experiment was a completely randomized design with a factorial combination of two treatments (AMF inoculation and soil Zn application) and five biological replicates per treatment, resulting in 30 wheat plants and 30 barley plants.

### Soil nutrient analysis

At physiological maturity (GS90), soil samples taken from the HCs placed into plant-free pots were oven-dried (105 °C for 48 h) and digested with *aqua regia* (following Zarcinas et al., 1996), for the determination of total soil Zn concentration. Additional soil samples were analysed for DTPA-extractable Zn [25]. Zinc concentrations in the digests and extracts was then determined by inductively-coupled plasma atomic emission spectrophotometry (ICP-AES, Spectroflame Modula, Spectro, Germany). Additionally, ^65^Zn activity in the digests was measured by γ-spectroscopy (1480 Wizard TM3®, Wallac, Germany).

### Harvesting and plant physiological analyses

Plants were destructively harvested at physiological maturity (GS90) after 73 and 83 days from transplanting for bread wheat and barley, respectively. Plants were cut at ground level and partitioned into grain, chaff and straw and subsequently oven-dried (60 °C for 48 h) for dry weight determination. Mean kernel dry weight was measured and number of kernels per spike and Spike Fertility Index (SFI) were calculated. The SFI is the ratio of number of kernels per spike to chaff dry weight per spike. Grain and straw samples were digested following Zarcinas et al. [53], prior to quantification of P and Zn concentrations by ICP-AES, and of plant ^65^Zn activity by γ-spectroscopy as described for soil (above). Due to the high γ emission measured in the pots (over 2 μS h^− 1^), the entire root systems could not be safely removed. Consequently, a sample of roots was collected from each pot by coring the soil (15 mm diameter and 120 mm depth). Roots were then carefully washed from the soil with RO water, and placed into 50% C_2_H_6_O (ethanol) prior to be cleared with KOH (10% *w/v*) [38] and stained with 5% ink in vinegar [44]. Stained roots were used for assessing AMF colonization by the gridline intersect method [28].

### Calculations

The activity of ^65^Zn in mock-inoculated plants did not differ from background activity. Therefore, we calculated for bread wheat and barley the mycorrhiza-mediated contribution to Zn uptake (mycorrhizal pathway of Zn uptake: MPU Zn) in grain and straw (as % and μg Zn) using the specific activity (SA) values of ^65^Zn in the soil and in the inoculated plants.

The SA in grain, straw and soil were calculated using the following equations (following [48]):

1a $$ \mathrm{Grain}/\mathrm{straw}\ \mathrm{specific}\ \mathrm{activity}=\frac{{}^{65}\mathrm{Zn}\;\mathrm{activity}\;\left(\mathrm{kBq}\;{\mathrm{g}}^{-1}\;\mathrm{grain}/\mathrm{straw}\;\mathrm{dry}\;\mathrm{weight}\right)}{\mathrm{Zn}\;\left(\upmu \mathrm{g}\;{\mathrm{g}}^{-1}\;\mathrm{grain}/\mathrm{straw}\right)} $$

1b $$ \mathrm{Soil}\ \mathrm{specific}\ \mathrm{activity}=\frac{{}^{65}\mathrm{Zn}\ \mathrm{activity}\ \left(\mathrm{kBq}\ {\mathrm{g}}^{\hbox{-} 1}\ \mathrm{soil}\right)}{\mathrm{Soil}\ \mathrm{DTPA}-\mathrm{extractable}\ \mathrm{Zn}\ \left(\upmu \mathrm{g}{\mathrm{g}}^{\hbox{-} 1}\ \mathrm{soil}\right)} $$

On the basis of Smith et al. [40] it was assumed that the AMF hyphal growth measured as hyphal length density in the extra HCs was similar to the growth in the whole pot. Therefore, the MPU Zn in grain and straw was calculated as follows:

2 $$ \mathrm{MPU}\ \mathrm{Zn}\ \mathrm{grain}/\mathrm{straw}\ \left(\%\right)=\frac{\mathrm{Equation}\ 1a}{\mathrm{Equation}\ 1b}\mathrm{x}\frac{\mathrm{Total}\ \mathrm{soil}\ \mathrm{weight}}{{}^{65}\mathrm{Zn}\ \mathrm{labelled}\ \mathrm{soil}\ \mathrm{weight}}\mathrm{x}100 $$

where total soil weight is the dry weight of the soil (1,400 g) in the whole pot and ^65^Zn labelled soil weight is the dry weight of the ^65^Zn-labelled soil in the HC (40 g).

3 $$ \mathrm{MPU}\ \mathrm{Zn}\ \mathrm{grain}/\mathrm{straw}\ \left(\upmu \mathrm{g}\right)=\frac{\mathrm{Grain}/\mathrm{straw}\ \mathrm{Zn}\ \mathrm{content}\ \left(\upmu \mathrm{g}\right)\ \mathrm{x}\ \mathrm{Equation}\ 2}{100} $$

The contribution of the DPU to Zn uptake in bread wheat and barley was calculated by the difference between total grain/straw Zn content (μg) and MPU Zn grain/straw content (μg). The total MPU contribution to Zn uptake (μg) as well the direct contribution of roots to Zn uptake (DPU; μg) were calculated as the sum of grain MPU/DPU Zn and straw MPU/DPU Zn. MPU Zn (%) was calculated as the ratio of MPU Zn uptake (μg) to total plant Zn content.

### Statistics and data analyses

Mycorrhizal colonisation of roots (% colonized root length), MPU and DPU Zn in grain and straw (% and μg), and total MPU and DPU Zn (% and μg) were analysed by one-way ANOVA, with Zn application used as fixed factor. We did not include the data from mock-inoculated plants in the statistical analysis of % colonized root length and MPU to Zn because the roots were not colonized and had no ^65^Zn activity. Above-ground dry weight, yield, yield components, and Zn and P content in grain and straw were analysed by two-way ANOVA, with AMF inoculation and Zn application the fixed factors. The one-way and two-way ANOVA data were ln- or arcsine-transformed when needed, to fulfil the assumptions of ANOVA. Where significant differences were found, the Tukey’s B post hoc test was performed to assess the differences among means. Mean and standard error value given in Tables and Figures represent non-transformed data. All data were analysed using SPSS 23.0 software (SPSS Inc., Chicago, IL).

## Additional file

Additional file 1: Supplementary materials to the manuscript including a Table of ANOVA outcomes, two Tables of plant response data (yield parameters and P contents), and one Figure depicting the experimental pot and hyphal compartment set-up. (DOCX 119 kb)

## Abbreviations

AMF: Arbuscular mycorrhizal fungi
RO: Reverse osmosis
