# Supplementary material for: The mycorrhizal pathway of zinc uptake contributes to zinc accumulation in barley and wheat grain
Source: BMC Plant Biol. 2019 Apr 10;19:133. doi: 10.1186/s12870-019-1741-y (PMC6456977; doi:10.1186/s12870-019-1741-y)
Supplement: Supplementary file 1 — Supplementary materials to the manuscript including a Table of ANOVA outcomes, two Tables of plant response data (yield parameters and P contents), and one Figure depicting the experimental pot and hyphal compartment set-up. (DOCX 119 kb) [file 12870_2019_1741_MOESM1_ESM.docx]

**Supplementary data**

| **Table S1.** *P*-values of one-way ANOVA evaluating the effect of three soil Zn levels on arbuscular mycorrhizal fungal (AMF) root colonization of bread wheat and barley inoculated with *Rhizophagus irregularis* and sampled at physiological maturity (Zadok's growth stage 90). | | |
| --- | --- | --- |
| **Factor** ^a^ | **AMF root colonization** | |
|  | **Bread wheat** | **Barley** |
| Soil Zn application ^b^ | **0.045** ^c^ | **0.009** |
| ^a^ Soil Zn application is used as a fixed factor. | | |
| ^b^ Soil Zn levels: 0, 20 and 75 mg kg^-1^ soil in the form of ZnSO_4_. | | |
| ^c^ In bold statistically significant values (*P* ≤ 0.05) (No of replicates=5). | | |

**Table S2.** Effect of AMF inoculation with *Rhizophagus irregularis* (+M *versus* mock inoculum, -M) and three soil Zn levels on arbuscular mycorrhizal fungal (AMF) root colonization and plant physiological traits of **bread wheat** sampled at physiological maturity (Zadok's growth stage 90).

| **Factors** | |  | **Aboveground biomass (g)** | **Chaff dry weight (g)** | **Spike fertility Index ^a^** | **Grain P content (mg P plant^-1^)** | **Straw P content (mg P plant^-1^)** |
| --- | --- | --- | --- | --- | --- | --- | --- |
| +M | Low Zn | mean | 2.74 | 0.48 | 77.78 | 3.62 | 1.62 |
|  |  | s.e. | 0.06 | 0.04 | 8.40 | 0.06 | 0.07 |
|  | Medium Zn | mean | 3.00 | 0.55 | 71.02 | 3.69 | 1.59 |
|  |  | s.e. | 0.06 | 0.08 | 12.60 | 0.12 | 0.14 |
|  | High Zn | mean | 2.75 | 0.62 | 54.38 | 3.44 | 1.50 |
|  |  | s.e. | 0.04 | 0.04 | 4.81 | 0.12 | 0.05 |
| -M | Low Zn | mean | 2.84 | 0.79 | 34.86 | 2.84 | 3.04 |
|  |  | s.e. | 0.06 | 0.08 | 4.68 | 0.16 | 0.12 |
|  | Medium Zn | mean | 2.91 | 0.80 | 39.82 | 2.96 | 2.66 |
|  |  | s.e. | 0.09 | 0.08 | 4.78 | 0.24 | 0.10 |
|  | High Zn | mean | 2.83 | 0.71 | 42.88 | 3.50 | 2.10 |
|  |  | s.e. | 0.04 | 0.15 | 6.88 | 0.18 | 0.06 |

| ^a^ Spike Fertility Index: No of kernels per spike/ chaff dry weight. |
| --- |

**Table S3.** Effect of AMF inoculation with *Rhizophagus irregularis* (+M *versus* mock inoculum, -M) and three soil Zn levels on arbuscular mycorrhizal fungal (AMF) root colonization and plant physiological traits of **barley** sampled at physiological maturity (Zadok's growth stage 90).

| **Factors** | |  | **Aboveground biomass (g)** | **Chaff dry weight (g)** | **Spike fertility Index** ^a^ | **Grain P content (mg P plant^-1^)** | **Straw P content (mg P plant^-1^)** |
| --- | --- | --- | --- | --- | --- | --- | --- |
| +M | Low Zn | mean | 2.82 | 0.60 | 40.96 | 2.10 | 3.20 |
|  |  | s.e. | 0.08 | 0.04 | 1.84 | 0.17 | 0.56 |
|  | Medium Zn | mean | 2.88 | 0.65 | 45.56 | 3.05 | 2.96 |
|  |  | s.e. | 0.11 | 0.10 | 6.69 | 0.74 | 0.52 |
|  | High Zn | mean | 3.01 | 0.60 | 57.12 | 3.05 | 2.74 |
|  |  | s.e. | 0.09 | 0.16 | 13.48 | 0.85 | 0.54 |
| -M | Low Zn | mean | 2.78 | 0.36 | 62.62 | 2.41 | 3.69 |
|  |  | s.e. | 0.09 | 0.06 | 13.21 | 0.20 | 0.30 |
|  | Medium Zn | mean | 2.83 | 0.52 | 57.34 | 2.24 | 4.52 |
|  |  | s.e. | 0.07 | 0.05 | 5.19 | 0.17 | 0.44 |
|  | High Zn | mean | 2.88 | 0.40 | 65.74 | 1.98 | 3.89 |
|  |  | s.e. | 0.04 | 0.05 | 5.68 | 0.14 | 0.17 |

^a^ Spike Fertility Index: No of kernels per spike/ chaff dry weight


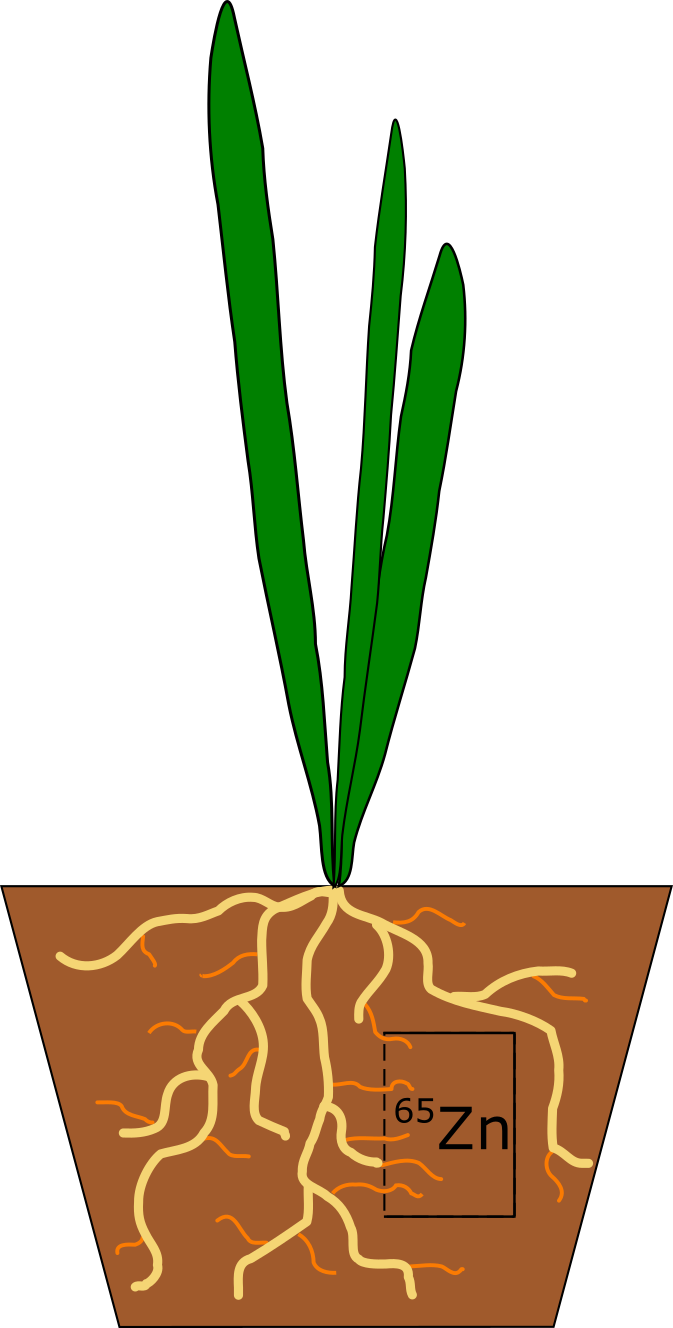


**Figure S1.** Diagram of the experimental pot set-up including a hyphal compartment (HC) containing soil mixed with radioactive ^65^Zn, accessible by the hyphae of the mycorrhizal fungi *Rhizophagus irregularis*, but not by the roots.
